# DNA recognition by *Escherichia coli* CbpA protein requires a conserved arginine–minor-groove interaction

**DOI:** 10.1093/nar/gkv012

**Published:** 2015-02-10

**Authors:** Kiran Chintakayala, Laura E. Sellars, Shivani S. Singh, Rajesh Shahapure, Ilja Westerlaken, Anne S. Meyer, Remus T. Dame, David C. Grainger

**Affiliations:** 1 Institute of Microbiology and Infection, School of Biosciences, University of Birmingham, Edgbaston, Birmingham B15 2TT, UK; 2 Leiden Institute of Chemistry, Gorlaeus Laboratories, Leiden University, Leiden, The Netherlands; 3 Department of Bionanoscience, Kavli Institute of Nanoscience, Delft University of Technology, Delft, The Netherlands

## Abstract

Curved DNA binding protein A (CbpA) is a co-chaperone and nucleoid associated DNA binding protein conserved in most γ-proteobacteria. Best studied in *Escherichia coli*, CbpA accumulates to >2500 copies per cell during periods of starvation and forms aggregates with DNA. However, the molecular basis for DNA binding is unknown; CbpA lacks motifs found in other bacterial DNA binding proteins. Here, we have used a combination of genetics and biochemistry to elucidate the mechanism of DNA recognition by CbpA. We show that CbpA interacts with the DNA minor groove. This interaction requires a highly conserved arginine side chain. Substitution of this residue, R116, with alanine, specifically disrupts DNA binding by CbpA, and its homologues from other bacteria, whilst not affecting other CbpA activities. The intracellular distribution of CbpA alters dramatically when DNA binding is negated. Hence, we provide a direct link between DNA binding and the behaviour of CbpA in cells.

## INTRODUCTION

**Figure 1.**
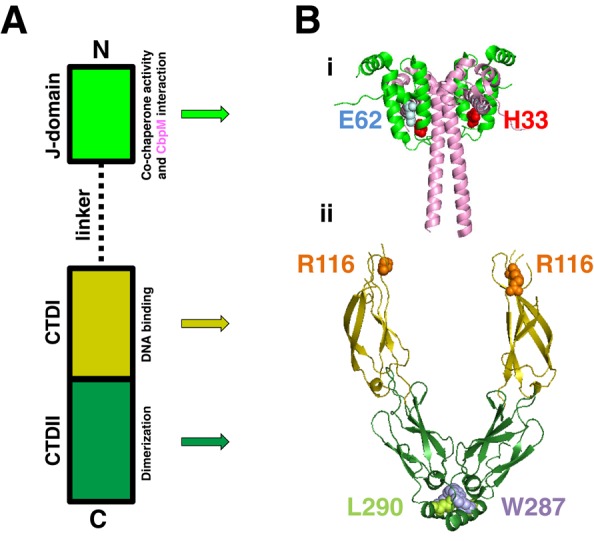
Organization of the CbpA protein. The figure shows schematic (**A**) and structural (**B**) representations of the CbpA protein. The J-domain is in light green, the linker is represented by a dashed line, C-Terminal Domain I (CTDI) is in mustard and C-Terminal Domain II (CTDII) is coloured dark green. Note that the CbpA J-domain interacts with the ‘modulator’ protein CbpM (pink). The function of each domain is indicted in panel (A). Amino acids involved in key interactions are highlighted in panel (B). CbpA side chain H33 interacts with CbpM residue E62. Amino acid R116 mediates DNA binding by CbpA and side chains W287 and L290 drive CbpA dimerization. Note that the relative positioning of the J-domain-CbpM complex, relative to the CTDI-II dimer, is purely speculative.

*Escherichia coli* curved DNA binding protein A (CbpA) was first isolated on the basis of its propensity to bind intrinsically curved, AT-rich, DNA molecules (1,2). It has subsequently been shown that CbpA is multifunctional, having both co-chaperone and DNA binding activities (3–6). Whilst the relationship between these activities is unknown it is clear that CbpA is a stress response protein; CbpA is produced during periods of starvation and DNA binding protects nucleic acids from damage (7). Protection results from the formation of protein–DNA aggregates similar in appearance to those formed by Dps (8). Conserved in many γ-proteobacteria, CbpA consists of three domains; the N-terminal J-domain is separated from two C-terminal domains (CTDI and CTDII) by a flexible linker (Figure 1A). Previously, Bird *et* *al*. characterized the function of each CbpA domain (3). The J-domain, a highly conserved feature of DnaJ-like co-chaperones, was shown to interact with DnaK (a chaperone) and CbpM (a CbpA inhibitor) but was dispensable for DNA binding, an activity that locates to the linker-CTDI region. Dimerization, a prerequisite for nucleic acid interactions, is mediated by CTDII (3,4,7). Hence, it is probable that dimerization correctly configures CbpA monomers to contact DNA. However, the identity of the precise DNA binding determinant remains unknown; the linker-CTDI region contains no obvious DNA binding motifs. Structural information is not available for the intact CbpA protein. However, data are available for the J-domain, a CTDI–CTDII fragment, and the J-domain in complex with CbpM (9–11). Hence, CbpM forms a dimer that can bind two copies of the CbpA J-domain (Figure 1Bi). The CTDI–CTDII dimer forms a 30 Å cleft (Figure 1Bii).

In combination with the structural studies described above molecular genetics has been used to reveal precise CbpA interaction surfaces (7,12). A basic patch on the surface of the J-domain, comprising amino acids R26, R30 and H33, interacts with CbpM side chains E64, T75 and E62 (11,12). Strikingly, this is the same surface of CbpA that contacts DnaK (11). Hence, this provides a molecular explanation for the ability of CbpM to inhibit co-chaperone activity. CbpM also blocks DNA binding by, and dimerization of, CbpA (3,4). This activity of CbpM is less well understood but is likely to involve the extended 50 Å α-helix, at the C-terminal end of CbpM, which is not involved in the J-domain interaction (Figure 1B). Dimerization of CbpA is mediated by a hydrophobic surface, comprising amino acid side chains W287 and L290, located on the same side of an α helix close to the C-terminus of CbpA (7,10).

In this work we have used a combination of genetics, biochemistry and cell biology to determine how CbpA binds to DNA. We show that, consistent with preferential recognition of AT-tracts, CbpA recognizes the minor groove of the double helix. This interaction is mediated by amino acid side chain R116, which sits at the boundary of the linker and CTDI. Replacement of R116 with alanine prevents DNA binding but does not disrupt CbpA dimerization or binding to CbpM. Importantly, we also show that R116 is conserved, in terms of sequence and function, in diverse bacteria. Using fluorescence microscopy, we observe that the intracellular distribution of CbpA is driven by DNA binding. Thus, we provide a link between the DNA-binding activity of CbpA and its function in the cell.

## MATERIALS AND METHODS

### Strains, plasmids and oligonucleotides

Bacterial strains and plasmids are listed in Table 1. Sequences of primers used to introduce mutations into *cbpA*, using QuickChange mutagenesis (Stratagene), are shown in Table 2. The different *cpbA*–mCherry alleles were incorporated into the *E. coli* MG1655 chromosome using gene doctoring (13). The donor plasmid was a derivative of pDOC-G with the gene encoding green fluorescent protein (GFP) replaced by a gene encoding mCherry. After digestion *in vivo*, via a nuclease provided by plasmid pACBSR, the donor plasmid generates a linear DNA fragment for recombination. Hence, this DNA fragment contains regions of chromosome homology upstream and downstream of the gene encoding mCherry. To replace *cbpA*, with *cbpA*–mCherry, the sequence of *cbpA* was used as the first homology region and 550 bp of sequence downstream of *cbpA* was used as the second. To introduce the R116A mutation *cbpA* R116A was fused to mCherry, creating pDOC–R*cbpA*KOR116, and recombination was driven by 500 bp of homology sequence upstream of the chromosomal *cbpA* and 550 bp of sequence downstream of *cbpA*. Strains were confirmed by colony polymerase chain reaction (PCR) before use.

**Table 1.** Strains and plasmids

Name	Description	Source
*Bacterial strains*		
BTH101	F′*cya*-99*ara*D139*galE*15*galK*16*rpsL*1(StrR)*hsdR*2*mcrA*1*mcrB*1	(19)
T7 express	*fhuA*2*lacZ*::T7gene1[*lon*]*ompTgalsulA*11R(*mcr*-73::miniTn10-TetS)	Invitrogen
	2[*dcm*]R(zgb-210::Tn10–TetS) *endA*1 D(*mcrC*-*mrr*)114::IS10	
MG1655Δ*cbpA*	Derivative of *Escherichia coli* MG1655 lacking the *cbpA* gene	This work
MG1655	Derivative of *E. coli* MG1655 encoding *cbpA*–mCherry	This work
*cbpA*–mCherry		
MG1655 R116A	Derivative of *E. coli* MG1655 encoding R116A *cbpA*–mCherry	This work
*cbpA*–mCherry		

*Plasmids*		
pKT25–CbpA	Encodes *Bordetella pertussis* CyaA T25 fragment fused to CbpA (Kan^R^)	(19)
pUT18–CbpA	Encodes *B. pertussis* CyaA T18 fragment fused to CbpA (Amp^R^)	(19)
pUT18–CbpM	Encodes *B. pertussis* CyaA T18 fragment fused to CbpM (Amp^R^)	(19)
pJ204	pUC derivative encoding AmpR	DNA2.0
pET21a*cbpA*	T7 Expression vector encoding native CbpA and derivatives	(7)
pDOC-G	Ecodes *sacB* gene and ampicillin resistance. Contains inserts for recombination that can be excised *in vivo* since they are flanked by I-SceI restriction sites.	(13)
pDOC–Rc*bpA*	pDOC-G derivative for inserting *cbpA*–mCherry into the chromosome	This work
pDOC–R*cbpA*	pDOC-G derivative for inserting R116A c*bpA*–mCherry into the	This work
KOR116A	chromosome of MG1655 *ΔcbpA*	
pDOC–K*cbpA*	Donor plasmid to knock out *cbpA*	This work
pACBSR	Carries the λ-Red and I-SceI endonuclease genes under the control of the *araBAD* promoter	(13)
pLER108	pACYC derivative encoding MalI-mCherry	L.E.S. PhD Thesis

**Table 2.** Oligonucleotides

Name	Description	Source
*Oligonucleotides for introducing Group I, II and III mutations into* cbpA		
GroupI up	5′-gcaatttaaccgtcagttccaccatggcgacggtgcggctgctgccgccgaagattttgacgata-3′	This work
GroupI down	5′-tatcgtcaaaatcttcggcggcagcagccgcaccgtcgccatggtggaactgacggttaaattgc-3′	This work
GroupII up	5′-cgatatcttctcgtcaattttcggtgcggctgccgcccagagccgtcaacg-3′	This work
GroupII down	5′-cgttgacggctctgggcggcagccgcaccgaaaattgacgagaagatatcg-3′	This work
GroupIII up	5′-tggcggtattcctcgaagaaacgcttgctgcggctgcgcgtaccatcagctataacct-3′	This work
GroupIII down	5′-caggttatagctgatggtacgcgcagccgcagcaagcgtttcttcgaggaataccgcca-3′	This work

*Oligonucleotides for introducing alanine substitutions into* cbpA		
R61A up	5′-ctgggaagtgttaagtgatgaacaagctcgcgctgagtat-3′	This work
R61A down	5′-atactcagcgcgagcttgttcatcacttaacacttcccag-3′	This work
H71A up	5′-ctgagtatgatcagatgtggcaagctcgcaacgatccg-3′	This work
H71A down	5′-cggatcgttgcgagcttgccacatctgatcatactcag-3′	This work
R72A up	5′-tgatcagatgtggcaacatgccaacgatccgcaatttaac-3′	This work
R72A down	5′-gttaaattgcggatcgttggcatgttgccacatctgatca-3′	This work
R79A up	5′-cgcaacgatccgcaatttaacgctcagttccaccatgg-3′	This work
R79A down	5′-ccatggtggaactgagcgttaaattgcggatcgttgcg-3′	This work
R110A up	5′-gcccgccagagcgctcaacgccccgc-3′	This work
R110A down	5′-gcggggcgttgagcgctctggcgggc-3′	This work
R116A up	5′-caacgccccgccacagccggccacg-3′	This work
R116A down	5′-cgtggccggctgtggcggggcgttg-3′	This work
R137A up	5′-aacgcttactgagcataaggctaccatcagctataacctg-3′	This work
R137A down	5′-caggttatagctgatggtagccttatgctcagtaagcgtt-3′	This work
K158A up	5′-tgatcgaacaggaaattccggcaacgctgaatgtgaagatcc-3′	This work
K158A down	5′-ggatcttcacattcagcgtggccggaatttcctgttcgatca-3′	This work
K163A up	5′-gaaaacgctgaatgtggcgatcccggcgggcgt-3′	This work
K163A down	5′-acgcccgccgggatcgccacattcagcgttttc-3′	This work
R173A up	5′-cgtcggcaatggtcaagccatccgtctgaaaggc-3′	This work
R173A down	5′-gcctttcagacggatggcttgaccattgccgacg-3′	This work
R175A up	5′-caatggtcaacgcatcgctctgaaaggccagggg-3′	This work
R175A down	5′-cccctggcctttcagagcgatgcgttgaccattg-3′	This work
K177A up	5′-aacgcatccgtctggcaggccaggggacgc-3′	This work
K177A down	5′-gcgtcccctggcctgccagacggatgcgtt-3′	This work
H199A up	5′-gatttgtggctggtgattgctattgcgccacatccgct-3′	This work
H199A down	5′-agcggatgtggcgcaatagcaatcaccagccacaaatc-3′	This work
R107A up	5′-cggtcagcatgccgcccagagccgtcaa-3′	This work
R107A down	5′-ttgacggctctgggcggcatgctgaccg-3′	This work
R112A up	5′-cccgccagagccgtcaagcccccgccac-3′	This work
R112A down	5′-gtggcgggggcttgacggctctggcggg-3′	This work
R130A up	5′-ggcatctcatgggatggctggacaggatcttgag-3′	This work
R130A down	5′-ctcaagatcctgttcagccatcccatgagatgcc-3′	This work

*Oligonucleotides for amplifying homology regions when constructing p-DOC derivatives*		
HRA*cbpA* up	5′-gcggaattcatggaattaaaggattattacgcc-3′	This work
HRA*cbpA*dwn	5′-gctggtacctgctttcccccaatctttacgtggatc-3′	This work
HRB*cbpM* up	5′-gcgctcgagatggctaatgttacggtgac-3′	This work
HRB*cbpM*dwn	5′-gcggctagcgcgcgttgtcgtcagtatgacagc-3′	This work
HRA*cbpA*KOup	5′-gctgaattcgcatttcctcaaattctttttctagtg-3′	This work
HRA*cbpA*KOdwn	5′-gctggtacccatagcgttatctcgcgtaaatc-3′	This work

*Oligonucleotides for cloning* cbpA *into pLER108*		
*cbpA* up *Nsi*I	5′-gcgatgcatgaattaaaggattattacgcc-3′	This work
*cbpA* down *Kpn*I	5′-gctggtacctgctttcccccaatctttacgtg-3′	This work

### Bacterial 2-hybrid analysis

β-galactosidase levels in BTH101 cells carrying derivatives of pKT25–CbpA and pUT18–CbpM or pUT18–CbpA were measured by the Miller method as described previously (7,14). Activities are shown in Miller units and are the average of three or more independent experiments. Cells were grown aerobically in MacConkey broth.

### Proteins and *in vitro* binding assays

The CbpA protein and derivatives were all purified using the protocol described extensively in previous work (7). For *p*-Hydroxyphenylglyoxal (HPG) modification 10 μM CbpA was treated with 10 mM HPG as described (15). Protocols for gel shift assays with CbpA are described by Chintakayala and Grainger (12). When required, Methyl Green or Netropsin were added to gel shift incubations prior to addition of CbpA. Protein and DNA concentrations used for all *in vitro* experiments are provided in the figure legends. At least two replicate gel shift experiments were done for each dataset presented.

### Tethered particle motion (TPM) analysis

Tethered particle motion (TPM) measurements were done essentially as described by Driessen *et al*. (16) using a micro-fluidic flow cell made by heat-sealing two thin cover glasses using Parafilm as a spacer (volume ∼40 μl). DNA fragments functionalized with digoxigenin (DIG) on one end and biotin on the other end (687bp, 43% GC content) were generated by PCR using functionalized primers (Eurogentec, Belgium). The glass surface was coated with 40 nM anti-DIG antibodies (Roche Diagnostics, Germany) dissolved in buffer A (33 mM Tris pH 7.9, 66 mM KAc, 1 mM dithiothreitol (DTT)) containing 0.2 mg/ml casein (Roche Diagnostics, Germany). After 20 min incubation the flow cell was rinsed with buffer A. Next, 0.2 nM of the 687 bp DNA dissolved in buffer A was flushed in and allowed to bind for 60 min. Finally, the flow cell was again rinsed with this buffer and streptavidin coated polystyrene beads with a diameter of 460 nm (Kisker Biotech, Germany) were introduced and allowed to bind for 45 min (0.1% v/v beads in buffer A containing casein). By rinsing with buffer A unbound beads were removed. In TPM experiments the Brownian motion of beads tethered to DNA was monitored by bright field microscopy (Nikon Diaphot 300, Japan) with a 100× oil immersion objective and imaged on a CCD camera (DCC1545M, Thorlabs, USA) at an acquisition rate of 25 Hz. The position of individual beads was tracked using a LabView program (National Instruments) as described by Laurens *et al*. (17). Tethers exhibiting a spherical scatter plot of x- and y-coordinates with a maximum eccentricity value of 1.3 were selected for further analysis. Root mean square motion (RMS) was computed using the formula $\sqrt{(x-\bar{x})^2+(y-\bar{y})^2}$. The *x* and *y* values are the coordinates of the bead at each instant of time and are the mean values calculated from bead positions over 40 s time. Bare DNA molecules and CbpA–DNA complexes were investigated in buffer B (20 mM Tris pH 7.0, 10 mM MgCl_2_, 120 mM KCl, 0.1 mM EDTA).

### DNA and protein sequence analysis

**Figure 2.**
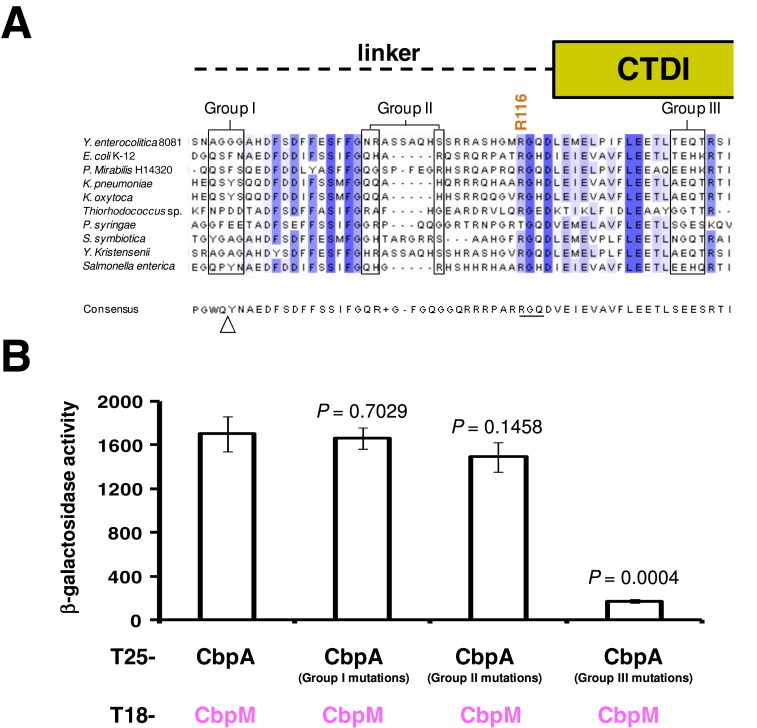
Effects of mutations in non-conserved regions of the linker-CTDI region. Panel (**A**) shows a selection of 10 CbpA linker-CTDI sequences from a larger alignment of 500 CbpA homologues. The CbpA homologs used in the alignment were the top five hundred hits in a microbial protein BLAST search using the *Escherichia coli* CbpA as the query sequence. The cartoon above the diagram indicates the location of amino acids with respect to the linker and CTDI. Residues previously implicated in DNA binding by Bird *et al*. are highlighted as Groups I, II and III (3). Conserved amino acids are highlighted in blue and a darker blue colour indicates better conservation. The sequence below the alignment is the consensus sequence derived from aligned CbpA homologues. The triangle indicates a position at which additional amino acids are inserted in many of the CbpA homologs in the full alignment. The underlined sequence is proposed to mediate DNA binding. Panel (**B**) shows a bar chart depicting results of BACTH assays performed to measure CbpA–CbpM interactions. Group I, II and III mutations are sets of alanine substitutions at the amino acid positions of *E. coli* CbpA boxed in Panel (A). The Student's *t*-test was used to calculate *P* by comparing β-galactosidase activity values generated by the T25–CbpA T18–CbpM interaction to those generated by CbpA derivates.

The alignment shown in Figure 2 was generated by searching the non-redundant protein sequence database using BLAST and *E. coli* K-12 CbpA as a query sequence. Note that this analysis identified 98 CbpA sequences from other *E. coli* strains that were ≥99% identical to the *E. coli* K-12 sequence. To avoid introducing bias these sequences were removed before generating the ‘consensus’ sequence shown in Figure 2A. To assess the DNA binding preferences of CbpA we utilized our previous map of CbpA binding across the *E. coli* genome using chromatin immunoprecipitation and a 43,450 feature DNA microarray (18). For each of the 43,450 probes we calculated the CbpA binding signal, the percentage AT content and the longest continuous sequence containing A and/or T. The probes were then divided into groups either on the basis of their AT content or by the length of the longest continuous AT tract. The average CbpA binding signal for each group of probes was then calculated.

### Fluorescence microscopy

Cells were grown in M9 minimal salts medium supplemented with 0.3% fructose and 0.1% casamino acids at 37°C for 24 h. 100 μl of culture was removed and washed 3 times with PBS before the pellet was resuspended in 20 μl PBS solution containing 5 μg/ml Hoechst 33258 and 40% glycerol. 5 μl was loaded onto poly-L-lysine coated slides and a cover slip applied. Slides were imaged using a Nikon Eclipse 90i microscope, Nikon Intensilight C-HGFI lamp, Hamamatsu ORCA ER camera (1344×1024 pixels, pixel size 6.45 μm) and Nikon Plan Apo VC 100x Oil immersion lens (Numerical Aperture 1.4), with a final optical magnification of 100x. A DAPI filter set was used for visualizing the Hoechst 33258 stained nucleoid and TxRed filter set for mCherry. Cells were also imaged using brightfield microscopy. Slides were prepared at room temperature and viewed within 30 minutes. Microscope images were analysed using NIS elements software (Nikon). At least 500 cells were measured for each condition in 3 biological repeats. For the analysis in Supplementary Figure S1 we first calculated the average fluorescence for the entire cell. This value was multiplied by the area of the cell to generate a value for total fluorescence.

## RESULTS AND DISCUSSION

### Assessment of amino acid substitutions known to disrupt DNA binding by CbpA

Previously, Bird *et* *al*. isolated individual CbpA domains to determine their function (3). Hence, DNA binding was shown to be a property of the linker-CTDI region. This study also examined the effect of clustered point mutations at various positions in the linker and CTDI. Three groups of mutations were found to disrupt DNA binding. Mutations in Group I (Q87A, S88A, F89A, N90A) and Group II (Q104A, H105A, R107A) fall in the linker region. Mutations in Group III (T133A, E134A, H135A, K136A) correspond to CTDI (Figure 2A). The initial aim of this work was to better understand these mutations and their role in DNA binding. As a starting point we generated an alignment of 500 CbpA homologues, with between 50% and 100% sequence identity, and examined sequence conservation in the linker-CTDI region. A selection of 10 sequences from the complete alignment is shown in Figure 2A. Note that a ‘consensus’ CbpA sequence, generated from aligned proteins, is also shown. Our expectation was that amino acids directly responsible for DNA binding should be conserved. However, on inspection of the alignment, it is apparent that none of the amino acids in Groups I, II or III are conserved. Moreover, whilst amino acids in Groups I and II are adjacent in *E. coli*, they are separated by amino acid insertions in other bacteria (Figure 2A).

Next, we determined whether the mutations characterized by Bird *et al*. were specifically defective for only DNA binding. The Bacterial 2-Hybrid (BACTH) system is a tool for measuring protein–protein interactions *in vivo* (19). Briefly, this system utilizes the Δ*cyaA E. coli* strain, BTH101, which is unable to produce cAMP. BTH101 can be transformed with plasmids pUT18C and pKT25 that encode two independently folding domains (T18 and T25) of the *Bordetella pertussis* adenylyl cyclase. When these plasmids are modified, so that they encode T18 and T25 fused to proteins that interact, cAMP production is restored. This induces expression of genes in the *lacZYA* operon, which can be quantified by measuring β-galactosidase activity. We previously showed that the BACTH system permits precise analysis of CbpA interactions with CbpM (7,12). Since the interaction with CbpM is mediated by the CbpA J-domain mutations in the linker-CTDI region should not influence CbpA–CbpM interaction unless they alter the structural integrity of CbpA (3). Thus, the three groups of mutations previously described by Bird *et al*. were introduced into the T25–CbpA fusion encoded by pKT25–CbpA. The pKT25–CbpA derivatives were used, along with pUT18–CbpM, to co-transform BTH101. Mutations in Groups I (*P* = 0.7029) and II (*P* = 0.1458) had no significant effect on the ability of CbpA and CbpM to interact. However, mutations in Group III (*P* = 0.0004) abolished the CbpA–CbpM interaction (Figure 2B). We note that Bird *et al*. reported mutations in Group I severely disrupted the co-chaperone activity of CbpA. Taken together, it seems unlikely that the amino acid side chains previously shown to disrupt DNA binding represent a precise nucleic acid recognition determinant; the amino acids are poorly conserved and some have complex affects on CbpA activity.

### CbpA–DNA interactions require access to the DNA minor groove

**Figure 3.**
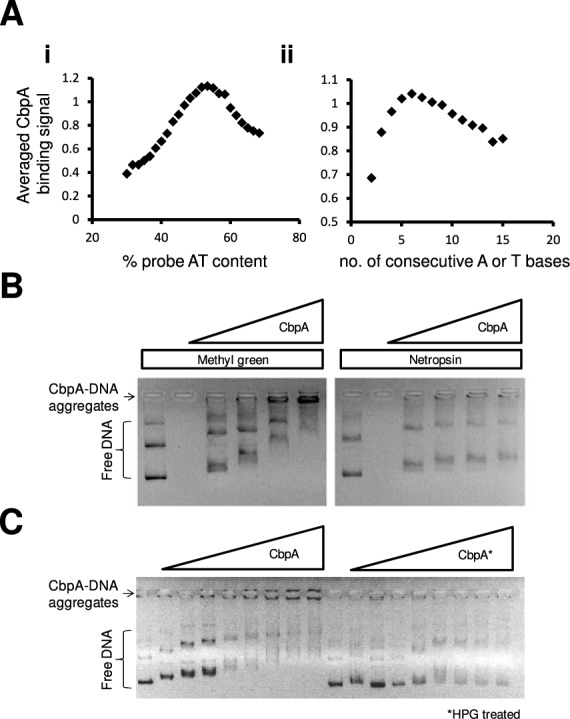
CbpA recognizes the DNA minor groove using an arginine side chain. Panel (**A**) shows two scatter plots that depict the relationship between CbpA binding and (i) DNA AT content and (ii) the length of AT-tracts. The CbpA DNA binding data was obtained from a previous analysis of CbpA binding across the *Escherichia coli* genome (17). Panel (**B**) shows two 1% (*w/v*) agarose gels on which CbpA binding to plasmid DNA was analysed in the presence and absence of 4 mM Methyl Green (a DNA major groove binding molecule) or 4 mM Netropsin (a DNA minor groove binding molecule). Bands corresponding to different topoisomers of the unbound plasmid are labelled as free DNA. Note that CbpA forms aggregates with DNA that accumulate in the wells of the gel. The final concentration of CbpA was between 2 and 8 μM. Panel (**C**) shows results of gel shift experiments where we compared the plasmid DNA (250 ng per lane) binding properties of CbpA before and after modification with 10 mM *p*-Hydroxyphenylglyoxal (HPG). Where added, the final concentration of CbpA was between 2 and 8 μM.

In an attempt to unravel the precise nature of the CbpA-DNA interaction we re-examined our previous analysis of CbpA binding across the *E. coli* genome (18). In particular, we focused on the properties of genomic regions that bound CbpA most tightly. To ascertain the relationship between DNA sequence and CbpA binding we grouped genomic sequences according to i) their percentage AT-content or ii) the length of the longest continuous AT-tract present. For each group of sequences we then calculated the mean CbpA binding signal. Consistent with previous observations this analysis confirmed that CbpA preferentially binds DNA with an above average AT-content (Figure 3Ai). Moreover, it is not sufficient for sequences to be AT-rich alone; AT-tracts of 6 base pairs in length are optimal for CbpA binding (Figure 3Aii). It is well-established that the minor groove is narrower at AT-tracts than other DNA sequences (20). Proteins that target AT-tracts frequently recognize this change in DNA conformation (21). We reasoned that CbpA may recognize DNA in this way. To test this hypothesis we examined the effect of Methyl Green and Netropsin on CbpA-DNA interactions. Methyl Green binds to the major groove of the double helix and Netropsin occupies the minor groove (22,23). Note that we previously showed CbpA forms aggregates with DNA that are detectable in gel shift assays as complexes in the wells of the gel (7,12). Thus, the formation of CbpA–DNA aggregates was used to measure effects of Methyl Green and Netropsin. The data show that Netropsin blocks aggregation of CbpA with DNA whilst Methyl Green does not (Figure 3B). Thus, efficient DNA binding by CbpA requires access to the DNA minor groove.

### CbpA–DNA interactions require arginine side chains

A recent compendium of structures in the Protein Data Bank (PDB) showed that 80% of all protein interactions with the DNA minor groove, at AT-tracts, are mediated by arginine or lysine (24). Of these interactions those involving arginine are most common. The reagent HPG reacts with exposed arginine side chains in proteins and modifies the guanidyl group (15). Thus, HPG treatment is a useful tool for determining if arginine side chains are involved in mediating the interaction between a protein and a given ligand. Briefly, in these experiments, the protein of interest is treated with HPG and excess HPG is then removed by gel filtration. The ligand binding activity of the protein is then tested. Thus, we examined the effect of HPG treatment on the ability of CbpA to bind DNA. Figure 3C shows the result of an EMSA experiment to monitor DNA binding by CbpA pre and post treatment with HPG. Treatment with HPG greatly reduces the ability of CbpA to bind DNA, as seen by a decreased intensity of DNA species that failed to run into the gel. This result is consistent with an important role for arginine side chains in DNA binding (Figure 3C). We conclude that CbpA interacts with the DNA minor groove and that this interaction requires an arginine side chain.

### CbpA residue R116 is evolutionarily conserved and essential for DNA binding

**Figure 4.**
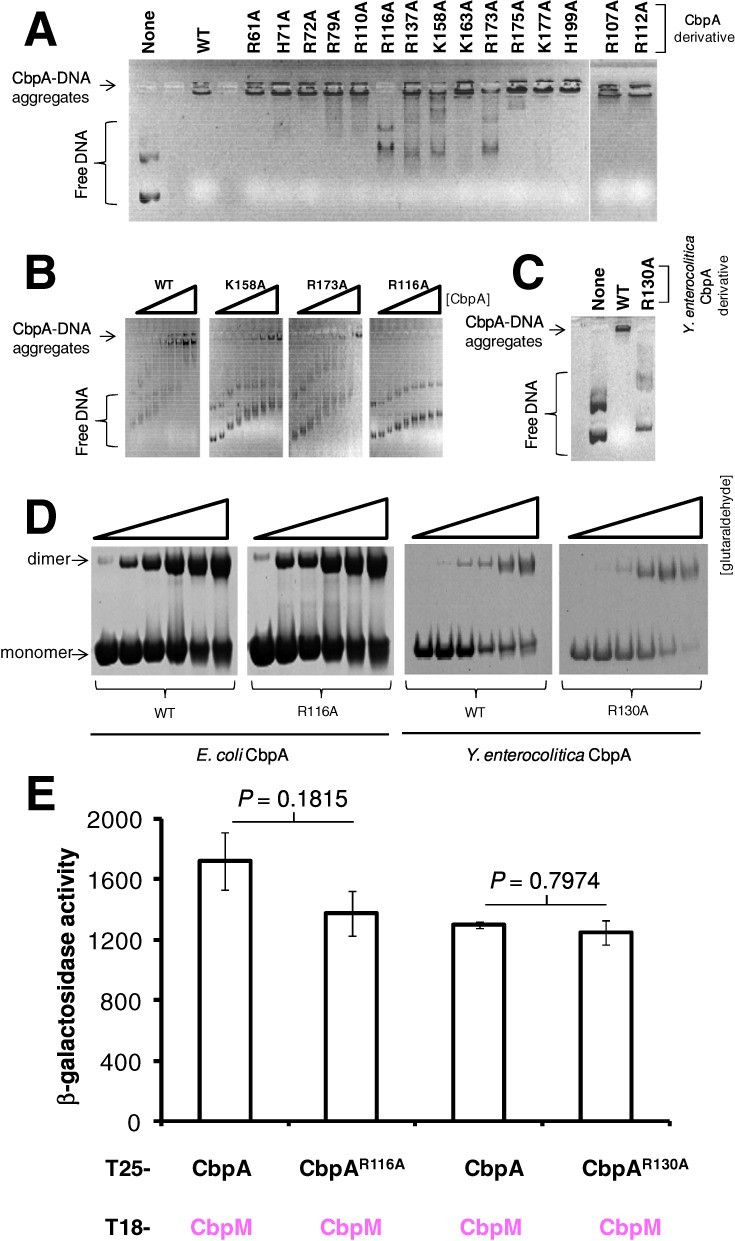
CbpA side chain R116 is a conserved DNA binding determinant. Panel (**A**) shows two 1% (*w/v*) agarose gels on which the binding of different CbpA (4 μM) derivatives to plasmid DNA (250 ng per lane) was analysed. Panel (**B**) shows DNA-aggregate formation by wild-type CbpA (and the R116A, K158A and R173A derivatives) in a set of titrations. Panel (**C**) is a gel shift experiment comparing binding *Yersinia enterocolitica* CbpA (4 μM) and the R130A derivative, to plasmid DNA (250 ng per lane). Panel (**D**) shows purified CbpA derivatives analysed on a sodium dodecylsulphate-polyacrylamide gel. Proteins (5 ng) were treated with between 0.0001% and 0.002% glutaraldehyde before loading. Monomeric and dimeric forms of CbpA are indicated. Panel (**E**) shows a bar chart depicting results of BACTH assays performed to measure CbpA–CbpM interactions. Different CbpA derivatives are indicated. The Student's *t*-test was used to calculate *P*.

To identify amino acid side chains responsible for DNA recognition we undertook an alanine scanning mutagenesis of CbpA. We focused on arginine, lysine and histidine (because of their positive charge) residues in the linker-CTDI region. Thus we generated 15 separate CbpA derivatives. These CbpA variants were purified and their ability to form aggregates with DNA was tested using gel shift assays. The data in Figure 4A show that only one substitution, R116A, completely abolished the ability of CbpA to form aggregates (i.e. complexes that failed to run into the gel). Only two additional point mutations (K158A and R173A) reduced the ability of CbpA to form aggregates with DNA by >50% (adjudged by quantifying the intensity of the band corresponding to the CbpA–DNA aggregate). We thus compared the R116A, K158A and R173A derivatives in precise titration experiments; only the R116A substitution abolished DNA aggregate formation (Figure 4B). Our alignment of CbpA homologs reveals that side chain R116 is conserved in 81% of the putative CbpA homologs (Figure 2A). In our alignment, one of the more divergent CbpA proteins is that from *Yersinia enterocolitica* subsp. 8081 (we refer to this as ^Ye^CbpA). In ^Ye^CbpA the key arginine residue falls at position 130 due to the insertion of extra amino acid sequences into the linker region. We purified the ^Ye^CbpA protein, and the R130A derivative, and investigated their DNA binding properties. As expected, the R130A derivative of ^Ye^CbpA is defective for DNA binding (Figure 4C).

### The CbpA R116A derivative can both dimerize and interact with CbpM

Our next goal was to determine if the R116A and R130A substitutions specifically induced defects in DNA binding or also hindered other CbpA activities. As we have demonstrated previously, CbpA dimerization can be monitored using *in vitro* glutaraldehyde cross-linking (7). In such experiments mutation of the CbpA dimerization interface prevents cross-linking of the CbpA dimer (7). Reassuringly, both CbpA R116A and ^Ye^CbpA R130A were indistinguishable from their cognate wild-type protein with respect to dimerization properties (Figure 4D). In an additional control experiment the BACTH system was used to measure interactions of the CbpA derivatives with CbpM. As expected, the alanine substitutions had no effect on interactions with CbpM (Figure 4E). We conclude that the CbpA R116A variant, and its equivalent from *Y. enterocolitica*, is specifically defective for DNA binding.

### Single molecule analysis of CbpA and the R116A derivative

**Figure 5.**
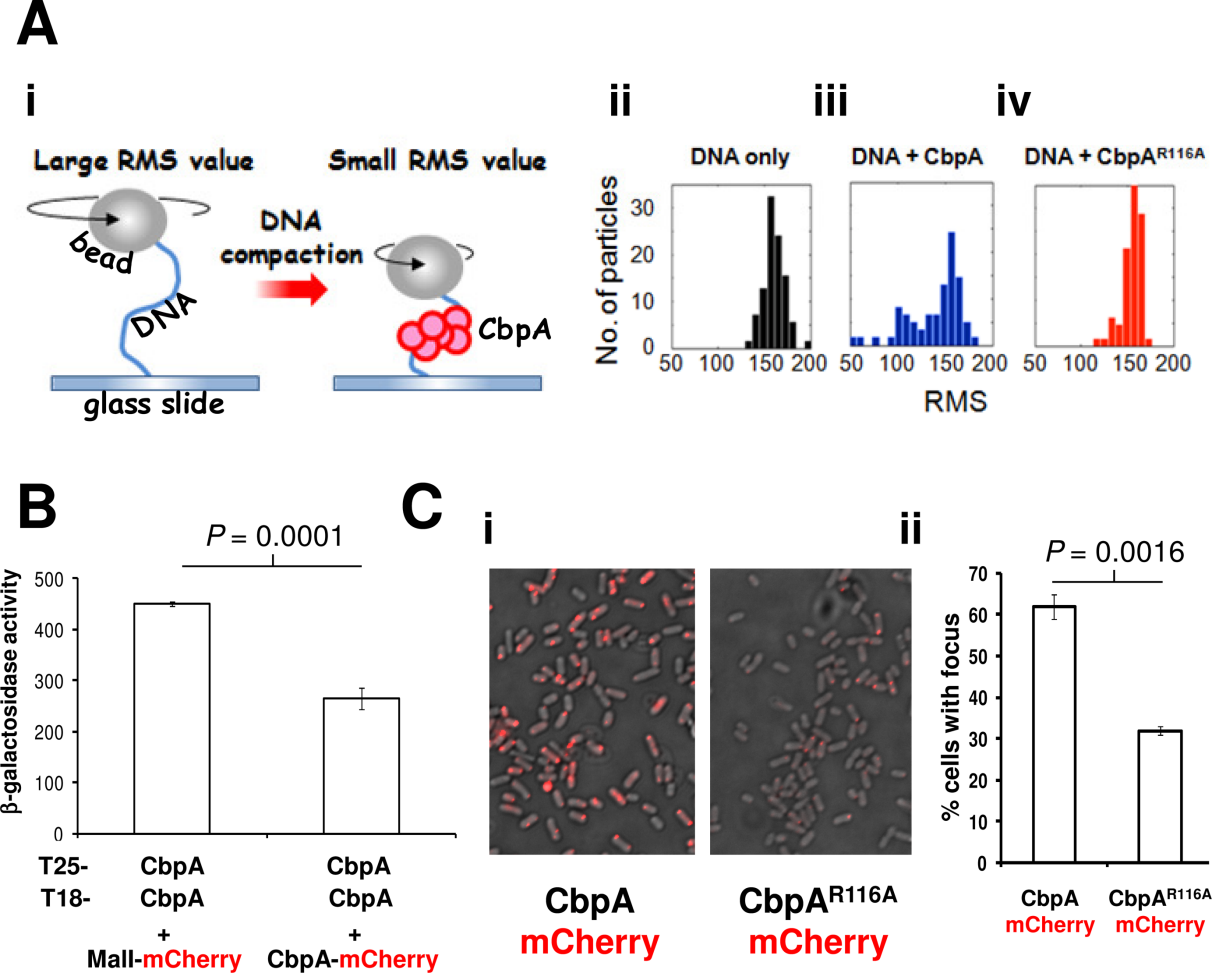
CbpA side chain R116 is required for aggregation of CbpA *in vitro* and *in vivo*. (**A**) Tethered particle motion analysis of CbpA–DNA complexes. (i) Schematic representation of the TPM experimental setup. Subsequent panels show histograms of RMS values for multiple particles tethered to (ii) naked DNA (iii) DNA in the presence of CbpA (iv) DNA in the presence of CbpA R116A. Incubations contained CbpA or the R116A derivative at a concentration of 750 nM. (**B**) Dimerization properties of a C-terminal CbpA–mCherry fusion protein. The bar chart depicting results of BACTH assays performed to measure CbpA–CbpA interactions. The Student's *t*-test was used to calculate *P* by comparing β-galactosidase activity values generated by the T25–CbpA T18–CbpA interaction in the presence and absence of CbpA–mCherry. (**C**) DNA binding controls the intracellular distribution of CbpA. (i) Fluorescence microscopy images of strain MG1655 *cbpA*–mCherry or the R116A derivative. (ii) A bar chart showing the mean number of CbpA foci for each CbpA derivative. Anova was used to calculate *P*.

CbpA aggregation with DNA has been demonstrated using gel shift assays, DNA protection assays and Atomic Force Microscopy (7,12). A limitation of gel shift analysis is that aggregates are difficult to detect if DNA is not saturated with CbpA. Thus, Tethered Particle Motion (TPM) analysis was used to monitor CbpA–DNA complexes more precisely (25). Figure 5Ai shows a schematic diagram describing TPM analysis. Briefly, a DNA molecule is tethered at one end to a glass slide and at the other end to a polystyrene bead. In solution, the bead is free to oscillate by Brownian motion. The only restriction on movement is the DNA tether. If the DNA tether is shortened, for example by aggregation of a DNA binding protein, the motion of the bead is restricted further. The benefit of this approach is that the properties of distinct populations of tethers (i.e. bound and unbound) are easily separable. Thus, formation of aggregates with DNA can be detected at extremely low CbpA concentrations. In these experiments the degree to which the bead can oscillate is expressed as a root mean squared (RMS) value of bead excursions. The average RMS value obtained using a naked DNA tether was ∼150 (Figure 5Aii). Addition of CbpA to incubations, at sub-saturating concentrations, resulted in the appearance of beads with restricted motion (RMS value ∼100) (Figure 5Aiii). Conversely, CbpA R116A did not alter the RMS value of the particles (Figure 5Aiv). Thus, CbpA interactions with DNA, and the effect of the R116A substitution, are detectable using different experimental approaches.

### The CbpA R116A derivative cannot form aggregates with DNA *in vivo*

In a final set of experiments fluorescence microscopy was used to visualize CbpA aggregation in live *E. coli* cells. To facilitate this, we generated a C-terminal CbpA–mCherry fusion protein. Note that the mCherry tag is located close to the CbpA dimerization determinant. Thus, before proceeding to the microscopy experiments, we tested dimerization of the fusion. When expressed ectopically in a BACTH experiment the fusion, if functional, should compete with T25–CbpA for binding T18–CbpA and *vice versa*. This should be detected as a reduced signal for CbpA dimerization in the BACTH assay. Fortuitously, the CbpA–mCherry fusion was functional for dimerization; interactions between T25–CbpA and T18–CbpA were reduced significantly (*P* = 0.0001) compared to a control experiment (Figure 5B). To visualize CbpA–mCherry in live cells the chromosomal copy of *cbpA* was modified to encode the mCherry fusion. Strain MG1655*cbpA*–mCherry encodes CbpA–mCherry under the control of native *cbpA* gene regulatory signals. Thus, the intracellular distribution of CbpA–mCherry, and the R116A derivative, was determined (Figure 5Ci). Consistent with previous reports using native CbpA we observed foci of CbpA (26). These foci occurred throughout the longitudinal axis of the cell but were most frequently found at the quarter cell position. Introduction of the R116A substitution resulted in a significant (*P* = 0.0016) decrease in the number of CbpA foci (Figure 5Cii). Logically, although the R116A substitution reduces the number of CbpA–mCherry foci, the total amount of CbpA–mCherry per cell (and hence total fluorescence) should not be altered. Hence, we also calculated total fluorescence per cell for each of the two stains shown in Figure 5Ci. As expected, the R116A substitution had no significant effect on total cellular fluorescence (*P* = 0.7874; Supplementary Figure S1). We conclude that the formation of intracellular CbpA foci is at least partly driven by DNA binding and aggregation.

## CONCLUSIONS

CbpA was initially isolated alongside the histone-like nucleoid structuring (H-NS) protein in a screen of *E. coli* cell extracts for proteins binding AT-rich DNA fragments (1,2). Unlike CbpA, the H-NS protein has been subjected to decades of intense scrutiny (27). Recently, the structure of H-NS in complex with DNA has been determined (28,29). This work revealed that H-NS recognizes the narrow minor groove associated with AT-tract DNA utilizing an arginine side chain on a surface exposed loop (28). Given that CbpA and H-NS were both isolated on the basis of their affinity for the same DNA sequences it is perhaps unsurprising that the mechanism by which they recognize DNA is also similar. In H-NS, the key DNA binding determinant is conserved and coincides with the amino acid motif Q/RGR (28). Interestingly, CbpA side chain R116 is part of a similar motif, RGQ, where both the arginine and glycine residues are highly conserved in CbpA homologs (underlined in Figure 2A). We propose that this motif represents the DNA binding surface of CbpA proteins in γ-proteobacteria. For *E. coli* CbpA we also detected effects on DNA binding of alanine substitutions at positions K158 and R173. Residue K158 is on the opposite face of CTDI with respect to R116. Conversely, R173 and R116 are located on the same surface of CbpA separated by ∼20 Å. Hence, it is possible that the two residues may interact with DNA simultaneously.

## SUPPLEMENTARY DATA

Supplementary Data are available at NAR Online.

SUPPLEMENTARY DATA
