# Supplementary material for: DNA recognition by Escherichia coli CbpA protein requires a conserved arginine–minor-groove interaction
Source: Nucleic Acids Res. 2015 Feb 10;43(4):2282–92. doi: 10.1093/nar/gkv012 (PMC4344490; doi:10.1093/nar/gkv012)
Supplement: SUPPLEMENTARY DATA [file supp_43_4_2282__index.html]

DNA recognition by Escherichia coli CbpA protein requires a conserved arginine–minor-groove interaction — SUPPLEMENTARY DATA 

# DNA recognition by *Escherichia coli* CbpA protein requires a conserved arginine–minor-groove interaction

## SUPPLEMENTARY DATA

**Files in this Data Supplement:**

- SUPPLEMENTARY DATA
