# Supplementary material for: DNA recognition by Escherichia coli CbpA protein requires a conserved arginine–minor-groove interaction
Source: Nucleic Acids Res. 2015 Feb 10;43(4):2282–92. doi: 10.1093/nar/gkv012 (PMC4344490; doi:10.1093/nar/gkv012)
Supplement: SUPPLEMENTARY DATA [file supp_gkv012_nar-03482-f-2014-File003.pdf]

Figure S1

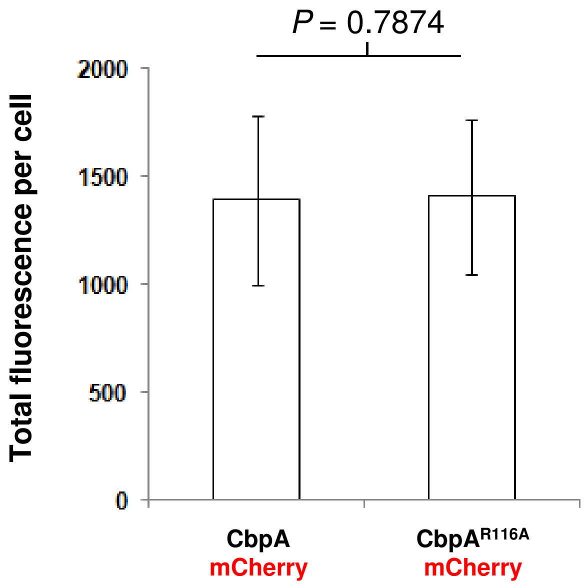

Figure S1: The R116A substitution does not alter the amount of CbpA mCherry per cell. A bar chart showing the averaged total fluorescence per cell due to CbpA-mCherry or the R116A derivative. Anova was used to calculate *P*.
